# Editorial: From Is to Ought: The Place of Normative Models in the Study of Human Thought

**DOI:** 10.3389/fpsyg.2016.00628

**Published:** 2016-04-28

**Authors:** Shira Elqayam, David E. Over

**Affiliations:** ^1^School of Applied Social Sciences, De Montfort UniversityLeicester, UK; ^2^Department of Psychology, Durham UniversityDurham, UK

**Keywords:** is-ought inference, meliorism, Panglossianism, descriptisim, rationality debate, normativism

Normative rules and regulations are everywhere we turn; they are, as Searle (2005) memorably called them, the glue that holds human society together. We stop at red lights and try (not always very successfully) to be fair and truthful in our personal and professional lives. We humans are the only species that internalizes normative rules (Carruthers, 2006), and feels shame and guilt when we violate them. Moreover, we humans are the only species capable of creating novel norms from scratch (Elqayam et al., 2015)—a species-specific, generative capacity no less extraordinary than the much-celebrated generative capacity to create novel sentences. It is not surprising, then, that normative rules feature so prominently in much of the psychology of higher mental processing—reasoning, decision making, and moral judgment. Normative rules dominate much of the great rationality debate, mainly in the form of the striking *normative-descriptive gap* (Stanovich and West, 2000). Human behavior often deviates from formal standards of rationality, such as classical logic and probability theory.

Can humans be said to be rational at all? The answer depends on whom you ask. *Meliorists* (Stanovich and West, 2000; Ariely, 2009; Kahneman, 2011) see the normative-descriptive gap as formidable, and a high level of human rationality as a rare phenomenon. From this viewpoint, being highly rational is like being a concert pianist—a great achievement and an unusual one. However, human rationality is amenable to education, and part of the Meliorist mission is to suggest how it might be improved. In contrast, *Panglossians* (Gigerenzer et al., 1999; Gladwell, 2007; Oaksford and Chater, 2009) see human rationality as a built-in evolutionary toolkit. Being rational, in this viewpoint, is the default—most of us are rational by dint of being human, just as most of us can all see and walk. If there is a gap between human behavior and any particular normative system, it is the normative system that is usually at fault.

As conflicting as they seem, Panglossianism and Meliorism nevertheless share some common ground. Both positions are *normativist:* They accept that rationality is measured by conformity to certain normative standards, while disagreeing, at least to an extent, on what those standards are, and how far the conformity exists. It is easy to see that identifying which normative standard is the right one would have far-reaching consequences for the Panglossians vs. Meliorists debate. Some normative standards may fit human behavior better than others, decreasing the normative-descriptive gap. In particular, the proponents of Bayesian rationality (Oaksford and Chater, 1998, 2007, 2009) suggested that probabilistic norms might provide a better fit to human rationality than norms derived from classical logic. However, arbitrating between normative standards is far from trivial. Elqayam and Evans (2011) criticized normativist theories (Panglossian and Meliorist alike) for trying to base this arbitration on empirical evidence, and so being in danger of committing the dubious inference from *is* to *ought*, considered a fallacy by many philosophers (Hudson, 1969; Pigden, 2010).

Normativist stances and practices are not, however, a universal phenomenon across the cognitive sciences. Linguists, for example, tend to be a lot less worried about violations of normative rules. Indeed, a tradition going back to De Saussure (1966) explicitly eschews normative concerns in favor of focusing on descriptive rules of language, the internalized ones that native speakers have in their heads. In the psychological literature on moral judgment, attitudes are more mixed, perhaps because the normative status of moral guidelines is far more controversial (although see Sunstein, 2005, for an attempt to derive moral norms from behavior).

The more recent position of *descriptivism* in the rationality debate (Elqayam and Evans, 2011; Evans and Elqayam, 2011; henceforth, collectively E&E) aims to follow in the footsteps of the Saussurean revolution in linguistics, proposing that the psychology of reasoning and decision making would be better off letting go of normative concerns altogether. Instead of measuring rationality by normative standards, the descriptivist position is that rationality should be measured by the achievement of personal goals. Evans and Over (1996) made a relevant distinction here between rationality_1_, measured by achieving one's goals, and rationality_2_, measured against some given normative standards. People can be rational_1_ without being rational_2_ and often are; and it is rationality_1_that is basic. Rationality_1_ is personal, contextualized, and relative, resulting in *grounded rationality* (Elqayam, 2012).

This Research Topic in *Frontiers in Cognitive Science* follows in the wake of a *Behavioral and Brain Sciences* treatment on normativism and descriptivism (E&E; and see commentaries there). In the current issue our aim was to widen the debate, allowing more space for discussion as well as empirical contributions. The result is a range of 23 articles from some 54 authors, on a diversity of topics from moral judgment to theory of mind. We divided the book into six main sections.

## The standard picture: normativist perspectives

This section encompasses contributions from the *standard picture* (Stein, 1996), that is, the classical normativist perspective. We start with Baron's introduction of the standard picture in the field of judgment and decision making (JDM). Because JDM is an applied field, normative models are necessary in order to evaluate behavior, with a view to ultimately improving it. In addition to normative and descriptive models, we also need *prescriptive* models, which specify how such improvement can be achieved.

Oaksford argues that rationality_1_ and rationality_2_ are inseparable. By Davidson's *charity principle*, rationality depends on normatively evaluating other people's behavior. Moreover, as logic and probability are compatible, there is no need to arbitrate between these normative standards. And, given that probabilistic norms are universal, the relativist concerns proposed in Evans and Elqayam (2011) and Elqayam (2012) are unjustified.

Hahn responds to three critiques of normative Bayesianism (Elqayam and Evans, 2011; Jones and Love, 2011; Bowers and Davis, 2012), arguing that the critique of Bayesian models is too general to be valid or useful. Specific accounts of reasoning, decision making, and argumentation provide counterexamples to the claim that Bayesian modeling is too flexible and thus un-falsifiable. Normative considerations have explanatory power that cannot be matched by descriptive accounts or process-level analysis.

Crupi and Girotto argue that what might appear to be debates about standards in classical reasoning and decision making are in fact nothing of the sort. Instead, the controversies are about mapping the stimuli or the responses onto specific norms, or a failure to identify what the relevant norm should be.

We conclude this section with Quintelier and Zijlstra's take on the is-ought problem itself. They suggest that an is-to-ought argument might actually be normative. They argue that inferring is to “ought” from “is” is best treated as a type of defeasible inference, rather than deductive inference. Such arguments should not be judged for their validity or soundness, but by appealing to the appropriate standards or evaluating defeasible arguments.

## In defense of soft normativism

Emerging from the debate in E&E, soft normativism is the view that, within boundaries, normative models have an important role to play in the psychology of reasoning and decision making, alongside more descriptivist considerations. Soft normativism comes with a moderate degree of relativism, which both contributions to this section accept. Stupple and Ball suggest that, as long as researchers are cautious of normativist research biases and focus on processing models, normative benchmarks have a role to play: they enrich our understanding of processing models, particularly in the Meliorist context of improving reasoning and judgment. Achourioti et al. draw on Searle's distinction between constitutive and regulative norms, arguing that normative models in reasoning and decision making are important for specifying both. The challenge in reasoning is to select the normative models appropriate to one's goals.

## Exploring normative models

The three contributions in this section focus on exploring and defending specific normative models. Markovits draws on Inhelder and Piaget (1958) to defend classical logic as the preferred normative model of rationality, arguing that the developmental evidence supports a notion of validity based on the existence of counterarguments. In contrast, the other two contributions support alternative normative models. Pothos and Busemeyer propose *quantum probability* as superior in explanatory power to classic (Bayesian) probability. Schwartenbeck et al. explore the *free energy principle*.

## Descriptivist perspectives

The contributors in this section accept the descriptivist position as departure point for their analysis. Evans takes descriptivism even further by arguing that the very notion of irrationality is problematic, depending as it does on an illusory presupposition that people are in conscious control of their minds and decisions. He concedes that deviations from normative standards can—and should—be construed as errors. However, these errors are merely evidence for limited capacity rather than irrationality.

Two further contributions explore normativism and descriptivism beyond the psychology of reasoning and judgment, ranging into Theory of Mind territory in philosophy and psychology. Iijima and Ota focus on the Knobe effect (Knobe, 2003), in which judgments of intentionality are affected by the perceived morality of the action. E&E argue that philosophers often misinterpret the Chomskyan distinction between competence and performance as normative, a muddle going back to Cohen (1981). Iijima and Ota accept this critique and extend it further, to criticize experimental philosophers for trying to draw unwarranted normative conclusions from the Knobe effect. Lastly, Wilkinson points out to the normativist stance in the psychological study of folk psychology. She argues that over-focus on questions of right and wrong in this study holds back research, and that more attention to the processing mechanisms underlying folk psychology would benefit the field.

## Evolutionary and ecological accounts

Much of the rationality debate in reasoning and decision making is cast in evolutionary, adaptationist, and ecological terms (Over, 2003), with approaches ranging from massive modularity (Cosmides and Tooby, 1994), through fast and frugal heuristics (Gigerenzer et al., 1999), to dual processing and beyond (Stanovich, 2004). This section presents three such accounts. We start with Brase's massively modular account. Like Achourioti et al. Brase rejects the one-size-fits-all notion of rationality. Instead, in each modular domain, a different type of rationality predominates, linked to the evolutionary goals set by the domain—such as self-protection, mate acquisition, and kin care, among others.

If Brase considers evolutionary pressures a source of rationality, Goel sees them as the opposite. Like Evans, Goel highlights the role of implicit, unconscious sources of thinking and deciding, but unlike Evans, he regards them as prime examples of irrationality. He argues that neither massive modularity nor dual processing accounts provide adequate explanations for the universal biological cues that trigger irrational behavior. Instead, he proposes an *adulterated rationality* account, in which a late-evolving rational system is often inadequately equipped to suppress instinctual, irrational responses.

Lastly for this section, Schurz presents a two-dimensional charting of cognitive success. First, he points to a parallel between the normative/instrumental rationality distinction and the *deontological*/*consequentialist* distinction in meta-ethics. In each, the basis of justification is either *a priori* normative obligation, or the utilitarian consequences of one's actions, respectively. The distinction, between *a priori* intuitions and *a posteriori* success, akin to Gigerenzer's ecological rationality, is orthogonal to the one between *logically-general* accounts and *locally-adaptive* ones. This two-dimensional mapping of rationality gives rise to novel research questions, supporting a dual account of cognition in which the selection of the appropriate cognitive tool takes center stage.

## Empirical reports

In this last and largest section we present a collection of empirical reports, with methods ranging from simulation to modeling. The *new paradigm* in psychology of reasoning (Elqayam and Over, 2013), a Bayesian and decision theoretic approach to reasoning, is strongly in evidence here. The section launches with two new paradigm studies of conditional reasoning, both using everyday causal conditionals such as “If oil prices continue to rise, then UK petrol prices will rise.” In both papers, descriptive models are held to provide a better fit for the data than models based purely on normative distinctions. Singmann et al. found that conformity, above chance, to coherence in conditional reasoning depends on the form of the inference. They advocate the *dual source* theory as a descriptive model. *This contribution was awarded the Best Student Paper Award of the Priority Program “New Frameworks of Rationality” for 2015*.

Trippas et al. used SDT (signal detection theory) to fit a large dataset of causal conditional reasoning with ROC (receiver operating characteristics) curves. They found that the descriptive theoretical modeling based on the difference between denial and affirmation inferences provided a better theoretical fit then the normative model based purely on inference validity. A debate follows this contribution, in which Singmann and Kellen dispute the SDT modeling in Trippas et al. arguing that they failed to make an unambiguous distinction between argument strength and response bias. Trippas et al. respond with a justification of their methods, and hold that their original point, that normative accounts are unreliable guides to conditional reasoning, remains in force.

Klaczynski uses individual differences measures to predict normative responding. Drawing on the classic methods of dual processing theories, he presents a large-scale individual differences study, showing that numeracy only predicted performance for participants who were both cognitively able and cognitively motivated.

We conclude this section—and the Topic—with two studies of judgment in social contexts. Wenmackers et al. simulation study identifies the individualistic nature of traditional approaches to human rationality, criticizing them for failing to take into account social-epistemic interactions between agents, such as information exchange. They test and support the Hegselmann–Krause model of epistemic interactions using computer simulations, which they argue are a useful bridge between normative models and descriptive results. Lastly, Gold et al. take the discussion (as Schurz does) into the realm of moral judgment. They criticize the artificiality of the trolley problems so widely used in moral judgment studies. Using more realistic scenarios, both in hypothetical contexts and operationalized in real life, they found that utilitarian responses were judged as more morally right for actors than for onlookers in traditional trolley problems, but reverse was true for a hypothetical game show context. When the game show was enacted in real life, the results reverted to the trolley dilemma pattern. They conclude with a discussion of the design choices in moral judgment experiments.

## Author contributions

All authors listed, have made substantial, direct and intellectual contribution to the work, and approved it for publication.

### Conflict of interest statement

The authors declare that the research was conducted in the absence of any commercial or financial relationships that could be construed as a potential conflict of interest.
